# The Epidemic of “Experts”

**Published:** 1898-01

**Authors:** 


### EDITORIAL DEPARTMENT.

F. E. DANIEL, M. D., Editor.
S. E. HUDSON, M. D., Managing Editor.
A. J. SMITH. M. D., Galveston, Associate Editor.

Published Monthly at Austin, Texas, by Drs. Daniel and Hudson. Subscription price $2.00 a year in advance.

Eastern Representatives: Messrs. Monihan & Fairchild, St. Paul Building, 220 Broadway, New York City.

Official organ of the West Texas Medical Association, the Houston District Medical Association, the Austin District Medical Society, the Brazos Valley Medical Association, the Galveston County Medical Society, and several others.

### THE EPIDEMIC OF "EXPERTS."

The recent epidemic from which the South suffered has confused medical men no little. There is no end to discussion and controversy as to its true nature. That thousands of cases of dengue occurred no one doubts; but there are many who say that it was all dengue that occurred in Texas. Surely no one denies that it was yellow fever at Edwards, Miss., and other points, and yet it all had the same origin, Ocean Springs, Miss., where in consequence of the inefficiency of the Marine Hospital officials yellow fever was carried from the national quarantine station on Ship Island. The occasion of the disease in Texas brought out some remarkable facts, foremost amongst which stands the astonishing fact that Texas is rich (or poor) in yellow fever "experts." Now, when we consider the fact that since 1869 there has been almost no yellow fever in Texas—no general prevalence; and excepting the local epidemic of 1873 at Columbus and some other points, which epidemic, by the bye, is said by one of the alleged experts of whom we are speaking, to have been not yellow fever, but a fever caused by dead fish in the river—and yet he bases his expert ability partly upon his experience with that epidemic—we may say there has been none. True, in 1876 and 1878 there were some cases of yellow fever at some of the inland towns. Now—we say—considering these facts, the question arises very naturally, where did the alleged experts who so emphatically denied the correctness of the diagnosis of yellow fever made by the State Health Officer and the representatives of the national government, acquire an experience sufficient to warrant their claim to expert knowledge of the disease? especially when we consider that some—many of them perhaps—were children, or at least not graduates, when the only chance to study the disease occurred in Texas, to-wit: 1869, at Galveston? If this question cannot be answered the alternative must be assumed as correct: that their pretentions to expert knowledge of yellow fever were not founded on any experience, and they are, therefore,—many of them,—pretenders only. What does Webster call a doctor who pretends to a knowledge he does not possess, based upon an experience he has not had?

The JOURNAL, as is well known, was founded some thirteen years ago, and its declared mission was, as a free lance, to denounce and expose all manner of sham, pretense and quackery in the medical profession, a mission it has filled, as its readers will testify,—without fear, and in some instances at cost of personal popularity and pecuniary loss. The JOURNAL had the temerity to expose the "Ghent's Hepatic Pill" scheme (dose one to three), worked by an ex-President of the Association, it will be remembered.

There is now another ex-President of the State Association who has made considerable notoriety in Texas in conneetion with the late yellow fever scare. The newspapers have represented him as a yellow fever expert, which title he has never disclaimed, but accepted as a matter of course. The papers represented him as going to Galveston from his inland home,—the scene of the dead-fish fever epidemic, which this expert has minutely described in Dowell's work on yellow fever—but which he never once says was yellow fever,—and—alone—nosing about back alleys "looking into the sanitary condition of the city;" to see perhaps, how far dead fish may be worked in as a probable factor in the fever of that place.

In our November number, we raised the question; where did Dr. Harrison graduate in yellow fever, and who conferred upon him the degree of expert? Our question remains yet unanswered, although we invited the doctor to answer it through the JOURNAL. Having a pretty good knowledge of the doctor's life history, gleaned from his autobiography, we did not see where he had ever possessed any opportunity to study yellow fever *since he had been a physician*, and hence the inquiry. We assert now that he has never in his life, since he has been an M. D., so far as we know, or have been able to learn, seen a case of yellow fever; and hence his pretentions are—pretentions only. He is no expert, and any efforts on the part of himself or friends to make it appear otherwise are simply absurd, in the face of his recorded history—his *auto*biography. Dr. Harrison's brother-in-law, an old gentleman named Towell, writes us that Dr. H. saw yellow fever in Hickman, Ky., in 1846. (Dr. Harrison was born in '26, and was then a youth of 20; so, *that* doesn't count.) He writes us also that Dr. H. saw lots of yellow fever in 1853, in New Orleans. (That was *twenty years*, exactly, before Dr. Harrison graduated in medicine, and was forty-four years ago; surely that is no legitimate claim.) The big epidemic in Texas, in 1867, was six years before Dr. H. got his diploma,—and surely *that* can't be counted. The first fever—at his own town—called by the other doctors there yellow fever (seeing the mortality was about 60 per cent.)—occurred in the summer of 1873, a few months after the doctor received his diploma; and, as, according to his own recorded belief this was not yellow fever, we ask again, when and where did Dr. R. H. Harrison, Sr., of Columbus, Texas,—an alleged yellow fever expert,—ever see a single case of yellow fever since he has been a physician in possession of a diploma? We ask for an answer; we challenge Dr. Harrison and his friends to show when and where he ever saw a single case of yellow fever, since he has been recognized as a graduate of medicine! As stated, he did not graduate until the spring of 1873 (Alabama Medical College), when he was 47 years of age; and he was elected President of the Texas Medical association in 1876. I have not the data at hand to show when he became a member of that Association, but it must have been some time before he was President, and as I have always understood that he was one of the original members who organized the Association away back in 1869, he must have joined before he had graduated, and the question naturally arises, if so, how did he get in?

In this connection it is amusing to read in the medical journals the following pronunciamento from the following supposed "experts." Some of these gentlemen are entirely unknown to the editor of this journal, notwithstanding he has rather an extensive acquaintance with the profession of the State, made during six years service as Secretary of the State Medical Association, and thirteen years as editor and publisher of the JOURNAL:

GALVESTON, TEXAS, Oct. 16, 1897.

Whereas, after having heard a full explanation of the prevailing epidemic now in Galveston, and also a thorough explanation of the courses and results of all the suspected cases of yellow fever, as diagnosed by Drs. Guiteras and Swearingen and some of the local physicians, we, the visiting physicians of the interior of Texas, resolve that we do not believe that there is a case of yellow fever in the State of Texas.

Resolved, further, That we do not believe there has been a case of yellow fever within the State of Texas within the past twelve months. While we believe that Drs. Guiteras and Swearingen, and others who have made diagnoses of yellow fever in Galveston and Houston were honest and conscientious in their diagnoses, the results have not borne out such diagnoses. J. F. Collier, M. D., Health Officer Montgomery County; J. E. Vann, M. D., Health Officer Trinity County; J. S. Wooters, M. D., Crockett; C. I. Holt, M. D., Kilgore; Lawrence Corley, M. D., Crockett; F. C. Woodward, M. D., Grapeland Board of Health; W. N. Hooper, M. D., Conroe; G. S. West, M. D., Palestine; W. G. Jameson, M. D., Palestine, Chief Surgeon International & Great Northern Railroad; J. L. Hall, Crockett; G. L. Davis, Trinity.

These gentlemen could with propriety certify that they had not seen a case in Galveston which they believed to be yellow fever; but in the name of consistency and common sense, upon what do they predicate the belief that there *has not been* a case of yellow fever in Galveston? By what did they judge? On what data did they rely to ascertain this astonishing belief? It is a stultification. This declaration on the part of the physicians of the far interior of the State, in face of the opinion of Dr. Guiteras, an expert of international fame; State Health Officer Swearingen, a yellow fever veteran; Prof. West, ex-Professor Practice of Medicine, Texas Medical College; Prof. J. W. McLaughlin, Professor Practice of Medicine, Texas Medical College; Dr. Truehart, of Galveston, who practiced through the epidemic of 1867, and a number of other experienced yellow fever physicians, places the signers in a ridiculous position.

